# Epiberberine: a potential rumen microbial urease inhibitor to reduce ammonia release screened by targeting UreG

**DOI:** 10.1007/s00253-024-13131-4

**Published:** 2024-04-08

**Authors:** Xiaoyin Zhang, Zhanbo Xiong, Yue He, Nan Zheng, Shengguo Zhao, Jiaqi Wang

**Affiliations:** State Key Laboratory of Animal Nutrition and Feeding, Institute of Animal Sciences, Chinese Academy of Agricultural Sciences, Beijing, 100193 China

**Keywords:** Urease inhibitor, Epiberberine, UreG, Nitrogen emission, Rumen

## Abstract

**Abstract:**

Rumen microbial urease inhibitors have been proposed for regulating nitrogen emission and improving nitrogen utilization efficiency in ruminant livestock industry. However, studies on plant-derived natural inhibitors of rumen microbial urease are limited. Urease accessory protein UreG, plays a crucial role in facilitating urease maturation, is a new target for design of urease inhibitor. The objective of this study was to select the potential effective inhibitor of rumen microbial urease from major protoberberine alkaloids in *Rhizoma Coptidis* by targeting UreG. Our results showed that berberine chloride and epiberberine exerted superior inhibition potential than other alkaloids based on GTPase activity study of UreG. Berberine chloride inhibition of UreG was mixed type, while inhibition kinetics type of epiberberine was uncompetitive. Furthermore, epiberberine was found to be more effective than berberine chloride in inhibiting the combination of nickel towards UreG and inducing changes in the second structure of UreG. Molecular modeling provided the rational structural basis for the higher inhibition potential of epiberberine, amino acid residues in G1 motif and G3 motif of UreG formed interactions with D ring of berberine chloride, while interacted with A ring and D ring of epiberberine. We further demonstrated the efficacy of epiberberine in the ruminal microbial fermentation with low ammonia release and urea degradation. In conclusion, our study clearly indicates that epiberberine is a promising candidate as a safe and effective inhibitor of rumen microbial urease and provides an optimal strategy and suitable feed additive for regulating nitrogen excretion in ruminants in the future.

**Key points:**

• *Epiberberine is the most effective inhibitor of rumen urease from Rhizoma Coptidis.*

• *Urease accessory protein UreG is an effective target for design of urease inhibitor.*

• *Epiberberine may be used as natural feed additive to reducing NH*_*3*_* release in ruminants.*

**Supplementary Information:**

The online version contains supplementary material available at 10.1007/s00253-024-13131-4.

## Introduction

Nitrogen, an essential element of all living organisms, plays a vital role in sustaining life on our planet (Kuypers et al. 2018). Meanwhile, some environmental problems caused by nitrogen pollution, including greenhouse gases, water, and soil pollution, are threatening human health (Bai et al. 2018; Fu et al. 2020). The livestock industry has been recognized as the largest source of NH_3_ emissions (Pandey and Chen 2021), contributing to approximately a third of global anthropogenic nitrogen emissions each year, of which rumen nitrogen emissions account for about 71% (Uwizeye et al. 2020). It has been reported that as much as 60 ~ 90% of the feed nitrogen can be excreted in urine and feces (Flachowsky and Lebzien 2006). Therefore, there is an urgent need to address these challenges by reducing nitrogen emission and cost of ruminant farming while improving nitrogen utilization efficiency.

Urea, a non-protein nitrogen feed, is commonly used as an ideal cost-efficient substitute of feed protein, which can be synthesized into microbial proteins to provide requirement for the host’s growth and milk synthesis through being broken down into ammonia by urease in rumen (Matthews et al. 2019; Reynolds and Kristensen 2008). However, high activity of urease decomposed urea into ammonia rapidly, and the rate of ammonia producing was faster than that of being utilized by rumen microorganisms, which was not synthesized into microbial protein and was excreted and escaped to the environment (Patra and Aschenbach 2018). In the progress, regulation of urease activity not only decreases nitrogen excretion and feed costs but also increases the efficiency of urea nitrogen utilization, and urease inhibitor is considered to be one of the most effective strategies.

Recently, plant-derived natural products have attracted much attention due to their natural origin, nontoxic and of low toxicity, chemically stable, and high bioavailability (Modolo et al. 2015; Kafarski and Talma 2018). Many natural inhibitors of microbial enzymes have been reported in various fields, such as human gut (Singhal and Rani 2022) and soil (Ye et al. 2022), which provided a good direction for the screening of urease inhibitor candidates. *Rhizoma Coptidis*, also known as *Huanglian* in Chinese, is the rhizome of *Coptis chinensis* Franch. It has been used to treat various inflammatory disorders and related diseases for a thousand years. Protoberberine alkaloids from *Rhizoma Coptidis*, such as epiberberine (Tan et al. 2017), coptisine (Li et al. 2018), and palmatine (Zhou et al. 2017), have been discovered to exert more potent inhibitory effect on *Helicobacter pylori* for the treatment of gastroenteritis. Tan et al. (2017) comparatively investigated the inhibitory potential against urease of 5 major alkaloids (berberine, epiberberine, coptisine, palmatine, and jateorhizine) based on the half inhibitory concentration (IC_50_) and found that epiberberine was the most promising urease inhibitor with the lowest IC_50_ value, and berberine, the most abundant alkaloid, was the weakest one among the 5 alkaloids. Li et al. (2016) also showed that berberine had little inhibitory activity against urease of *Helicobacter pylori*. By using antibacterial study and measuring urease activity of *Helicobacter pylori* in vivo, Li et al. (2018) showed that coptisine exerted the strongest inhibitory effect among the 5 major protoberberine alkaloids. In addition, our team demonstrated that coptisine inhibited the activity of rumen microbial urease and improved urea utilization efficiency of ruminants (He et al. 2022). To identify the most inhibitory potential alkaloids in rumen, we evaluated the inhibitory effects of the 5 major protoberberine alkaloids from *Rhizoma Coptidis* through multiple aspects.

Urease is composed of apo-urease and accessory urease proteins containing UreD (UreH), UreE, UreF, and UreG. Apo-urease commonly recognized as a target for the design of urease inhibitors through either competitively combining with the active sites of urease or mimicking urea, the substrate of urease (Kappaun et al. 2018). However, the active sites were deeply buried in the urease (Jabri et al. 1995), and the substrate of urease was highly specific (Ha et al. 2001), which makes it very challenging for developing effective urease inhibitors. Yang has been studying urease structure and urease maturation process and found that accessory protein UreG can be used as a new target for the design of urease inhibitor (Yang et al. 2018). During the process of urease maturation, nickel was transferred from UreE to UreG. Subsequently, nickel delivery from Ni-UreG to apo-urease was triggered by the formation of supercomplex apo-urease/UreFHG in *Helicobacter pylori*. Importantly, nickel insertion into the active center for activate urease was dependent on GTP hydrolysis by UreG and conformational changes of UreG (Fong et al. 2013; Yuen et al. 2017). Therefore, UreG played a key role in facilitating urease maturation, and inhibiting the UreG GTPase activity or disrupting the function of UreG to inactivate urease might be considered as a superior strategy for developing effective inhibitors.

The aim of the research was to select the potential effective inhibitor of rumen microbial urease from these 5 protoberberine alkaloids in *Rhizoma Coptidis* and their alkaloid hydrochlorides by targeting UreG. Two promising alkaloids were preliminary identified by measuring the effect on GTPase activity of UreG. Further experiments, including kinetic study, the effects of two alkaloids on the combination of nickel towards UreG and the secondary structure of UreG, as well as inhibitory sites of alkaloids to UreG, were conducted to reveal the inhibition mechanisms and to compare the inhibitory potential. Combined with the results of urease activity, the most promising inhibitor of rumen microbial urease were identified, and the inhibitory efficacy was further confirmed using the experiment of ruminal microbial fermentation. Overall, the study provides an effective inhibitor of rumen microbial urease by targeting UreG, promotes the application of plant-derived natural products in ruminant.

## Materials and methods

### GTPase activity of UreG

UreG (GenBank ID: MN660252) is an accessory protein from the predominant urease obtained from the rumen microorganisms of dairy cows, the preparation of UreG, and the measurement of UreG GTPase activity as described previously (Zhang et al. 2020a). Briefly, the gene of UreG was cloned into pASK-IBA5C plasmid and subsequently transformed into *Escherichia coli* BL21 (DE3) cells for protein expression, in which the expression plasmid was induced by anhydrotetracycline. The final UreG (purity > 95%) was obtained by purifying crude cell lysates using Strep-Tactin beads (Beaver, Suzhou, China). UreG GTPase activity was measured through the Malachite Green Phosphate Assay Kit according to the manufacturer’s instructions (Sigma-Aldrich, MI, USA). The inhibition rate of alkaloids (Target Molecule Corp., MA, USA) to inhibit UreG GTPase activity was calculated by the following formula:

$$\mathrm{Inhibition\;rate }\left(\%\right)=\left(1-\frac{A-B}{C-D}\right)\times 100$$

where *A* represents the absorbance value of UreG GTPase activity with alkaloid, *B* represents the absorbance value of GTPase activity of inactivated UreG with alkaloid, *C* represents the absorbance value of UreG GTPase activity without alkaloid, and *D* represents the absorbance value of GTPase activity of inactivated UreG without alkaloid. Each measurement was performed in triplicate.

IC_50_ value for alkaloid against UreG GTPase activity was calculated using the GraphPad Prism (v.8.0.1) software (GraphPad Software Inc., CA, USA), in which various concentrations of alkaloids (0, 6.25, 12.5, 25, 50, and 100 µM) and standards were prepared at the same reaction conditions, the GTPase activity of inactivated UreG without alkaloid was set as 0, and the GTPase activity of UreG without alkaloid was set at 100%.

### Kinetic studies

The kinetics assay of UreG by alkaloid was carried out by Michaelis–Menten equation, and the values of Michaelis–Menten constants (*K*_m_ and *V*_max_) were calculated from the Lineweaver–Burk plots through the software of GraphPad Prism. The inhibition with various concentrations of alkaloids (0, 15.6, 62.5, and 250 µM) was measured at different concentrations of GTP substrate (0, 12.5, 25, 50, 100, and 200 µM). Furthermore, the type of enzyme inhibition, including competitive inhibition, uncompetitive inhibition, non-competitive inhibition, and mixed inhibition, was revealed based the trend of *K*_m_ and *V*_max_ changing with the concentration of alkaloid.

### Isothermal titration calorimetry (ITC) measurements and circular dichroism (CD) spectroscopy

The inhibition mechanism of UreG by alkaloid was explored using ITC and CD spectroscopy, to reveal the effects of alkaloid on the combination of nickel towards UreG and the secondary structure of UreG. ITC measurements were carried out with an AutoITC200 microcalorimeter (GE, MA, USA) as described previously (Zhang et al. 2020a). Briefly, 27 µM UreG was titrated with 1500 µM NiSO_4_ in the present of 238 µM alkaloid. The titration curve was fitted with the one-site binding model, and the final data was analyzed by ITC Analysis Module in the software of Origin 7.0 (OriginLab, MA, USA).

The CD spectra of UreG were recorded using Chirascan Plus spectrometer (Applied Photophysics Ltd., Surrey, UK) equipped with a 1-mm quartz cell, at measurement range between 190 and 260 nm, with 1 nm data pitch and bandwidth. To reduce interference from the background solution, 233 µg/mL UreG and 88 µM alkaloid were prepared using distilled water. The final CD spectrum of UreG was obtained by subtracting the background spectrum (distilled water or alkaloid) from the sample spectrum. The secondary structure proportions (α-helix, β-sheet, β-turn, and random coil) in UreG were calculated using CDNN (v.2.1) software (Applied Photophysics Ltd., Leatherhead, UK).

### Molecular docking studies

Binding characteristics of UreG and alkaloid were analyzed performing molecular docking studies. UreG were modeled using the SWISS-MODEL server, and *Kp*UreG (PDB ID: 5XKT) was chosen as the template because of high sequence identity of 77.39%. The 3D structures of berberine chloride and epiberberine were downloaded from PubChem website (https://pubchem.ncbi.nlm.nih.gov/). Molecular docking of UreG and alkaloid was performed using AutoDockTools (v.1.5.6) (Scripps, CA, USA), in which the UreG was considered as a rigid structure receptor, and alkaloid was considered as a flexible molecule. The steps and parameters during molecular docking were consistent with those of previous article (Zhang et al. 2021). A total of 10 poses were obtained for each alkaloid, and the best docking pose with the lowest energy was further analyzed using PyMol molecular graphics system.

### Urease activity

Ruminal microbial crude protein was collected by ultrasonication and centrifugation following a previously established method (Zhang et al. 2020b). The urease activity of ruminal microbial crude protein was determined based on the amount of ammonia decomposed by urea through a modified phenol/hypochlorite reaction method (Krallmann-Wenzel 1985), in which the urease activity without alkaloid and urea was set at 0, and the urease activity without alkaloid was set at 100%. The inhibition rate of alkaloid to inhibit urease activity was calculated by the following formula:

$$\mathrm{Inhibition\;rate }\left(\%\right)=\left(1-\frac{A-B}{C-D}\right)\times 100$$

where *A* represents the absorbance value of urease activity with alkaloid, *B* represents the absorbance value of urease activity with alkaloid but without urea, *C* represents the absorbance value of urease activity without alkaloid, and *D* represents the absorbance value of urease activity without alkaloid and urea. Each measurement was performed in triplicate.

### Ruminal microbial fermentation

The study of ruminal microbial fermentation consisted of a control group and 3 epiberberine treatment groups, each containing 70 mL fresh rumen fluid and 140 mL anaerobic medium (Zhang et al. 2020c). Epiberberine treatment groups with the final concentration of 2, 20, and 200 μM were pre-incubated in an anaerobic chamber at 39 °C for 15 min. Immediately after inoculation, 10 mL aliquots of mixed solution were transferred into Hungate tubes, urea with a final concentration of 15 mM was added into each tube. Three tubes were immediately collected from each group as 0 h samples; the others were sealed with rubber stoppers and cultured in an incubator. Samples were collected at 1, 2, 4, 8, and 12 h of incubation, with 3 tubes in each group.

The collected tubes were immediately placed on ice to slow down fermentation; 400 μL of sample solution was mixed with 800 μL metaphosphoric acid solution (25% w/v) and centrifuged at 12,000 × *g* for 5 min. The supernatant was collected for the detection of NH_3_ and urea. NH_3_ content was measured by a modified phenol/hypochlorite reaction method. Urea concentration was determined using the diacetyl monoxime method kit following the manufacturer’s instructions (Jiancheng Bioengineering, Nanjing, China).

### Statistical analysis

The data of urease activity and the binding nickel to the UreG were analyzed by one-way analysis of variance (ANOVA) in SPSS v.25.0 software (IBM, NY, USA). Statistical significance *P* < 0.05 was accepted.

## Results

### Screening of urease inhibitor from main alkaloids in Rhizoma Coptidis

**Fig. 1:**
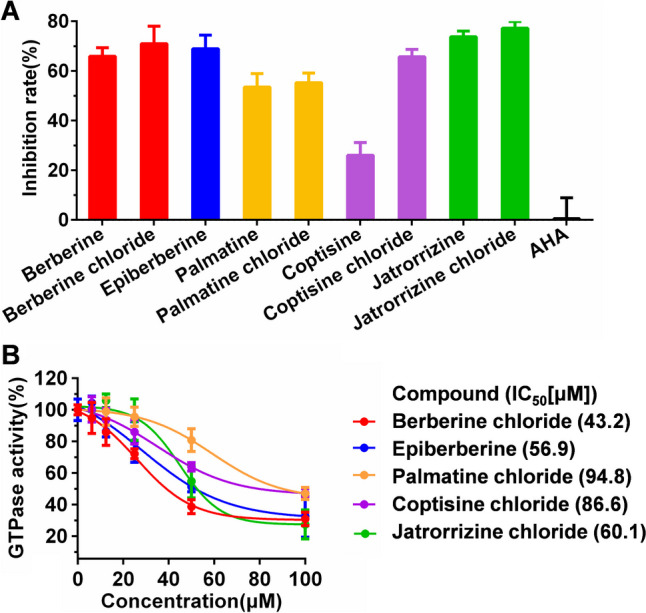
Screening of UreG inhibition of major protoberberine alkaloids in *Rhizoma Coptidis*. **A** Inhibition rate of protoberberine alkaloids and acetohydroxamic acid (AHA) on UreG GTPase activity. Results are desired as means ± SD of triplicate tests. **B** Effect of epiberberine and alkaloid hydrochlorides on UreG GTPase activity. The IC_50_ value against UreG GTPase activity was calculated at various concentrations of alkaloids (0, 6.25, 12.5, 25, 50, and 100 μM). Each point represents means ± SD of triplicate tests

Major alkaloids and alkaloid hydrochlorides in *Rhizoma Coptidis* were used to screen potential urease inhibitors by measuring the inhibition rate of UreG GTPase activity. As shown in Fig. 1A, except for coptisine, the inhibition rates of other alkaloids were higher than 50%. Interestingly, the inhibition rates of alkaloid hydrochlorides were slightly higher than alkaloids themselves. In addition, the commercially available urease inhibitor of acetohydroxamic acid (AHA) had little effect on UreG GTPase activity. Furthermore, alkaloid hydrochlorides and epiberberine were evaluated for their inhibitory capability against UreG by IC_50_ value (Fig. 1B). These alkaloids showed concentration-dependent inactivation activity with IC_50_ values ranging between 43.2 and 94.8 μM. Out of the 5 alkaloids, berberine chloride showed more potency with IC_50_ value of 43.2 μM lower than that of others. Epiberberine was the second most potent inhibitor with IC_50_ value of 56.9 μM. So, we selected the top two compounds with higher inhibitory potential for further analyzed.

### Kinetic study of UreG inhibition by alkaloids

The kinetic analyses in different concentrations of alkaloids and GTP substrate are shown in Table 1, Table 2, and Fig. S1. The reaction velocity gradually enhanced with the increase of substrate GTP concentration and decreased with the increase of alkaloid concentration (Fig. S1). For berberine chloride, with the increased concentration of inhibitor, *V*_max_ value gradually decreased, and *K*_m_ value decreased first and then increased, indicating that berberine chloride was a mixed-type inhibitor for UreG (Table 1). For epiberberine, both the values of *V*_max_ and *K*_m_ gradually decreased after adding different concentrations of epiberberine, suggesting an uncompetitive inhibition type towards UreG (Table 2).

**Table 1:** Kinetic parameters of UreG GTPase activity in the presence of different concentrations of berberine chloride (0, 15.6, 62.5, and 250 μM)

Concentration (μM)	Equation	*R* square	*V*_max_	*K*_m_
250	*y* = 6.786*x* + 0.04787	0.9929	20.9	141.8
62.5	*y* = 2.219*x* + 0.04726	0.9856	21.2	47.0
15.6	*y* = 1.811*x* + 0.03897	0.9984	25.7	46.5
0	*y* = 2.120*x* + 0.02850	0.9965	35.1	74.4

**Table 2:** Kinetic parameters of UreG GTPase activity in the presence of different concentrations of epiberberine (0, 15.6, 62.5, and 250 μM)

Concentration (μM)	Equation	*R* square	*V*_max_	*K*_m_
250	*y* = 2.510*x* + 0.09706	0.9637	10.3	25.9
62.5	*y* = 2.348*x* + 0.05610	0.9855	17.8	41.9
15.6	*y* = 2.140*x* + 0.03307	0.9883	30.2	64.7
0	*y* = 2.120*x* + 0.02850	0.9965	35.1	74.4

### Alkaloids inhibited nickel binding to UreG

**Fig. 2:**
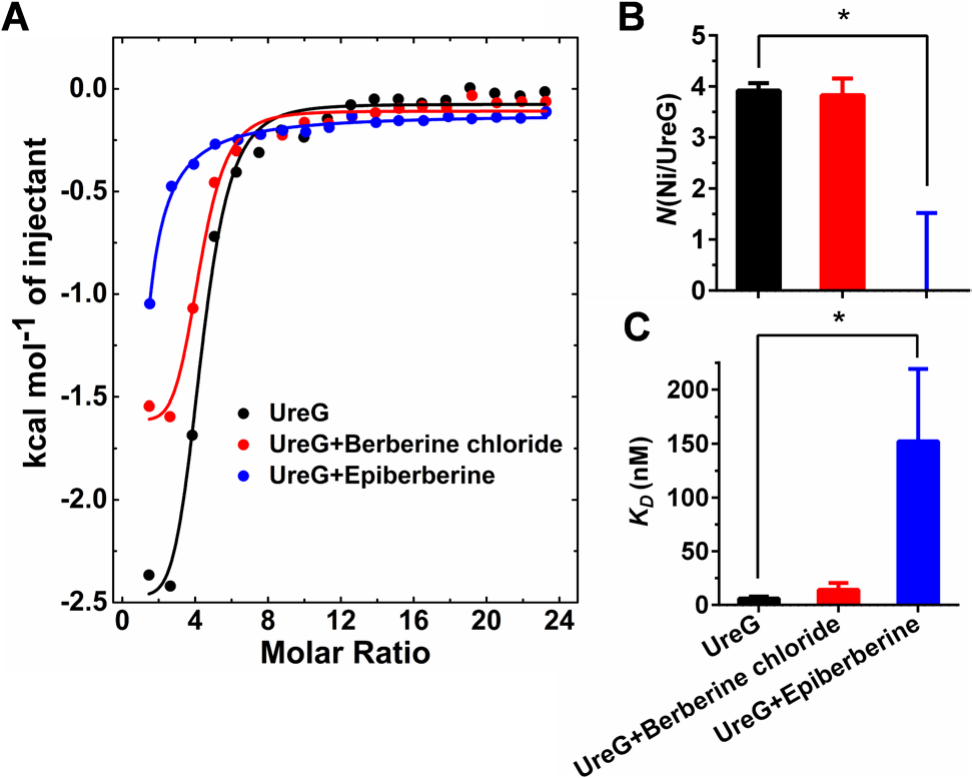
Effect of berberine chloride and epiberberine on the combination of nickel towards UreG. **A** Isothermal titration calorimetry enthalpograms of nickel binding to UreG in the absence or presence of alkaloids. Titration data are presented as colored solid circle and fit as corresponding solid lines. **B** Effect of berberine chloride and epiberberine on molar ratio (*N*) of nickel and UreG. **C** Effect of berberine chloride and epiberberine on equilibrium dissociation constant (*K*_*D*_) between nickel and UreG. Results are desired as means ± SD of triplicate tests. * represents significant difference between groups

To understand the possible inhibition mechanism of different alkaloids, the changes of nickel binding to UreG were examined using ITC experiments. Urease is a nickel-dependent enzyme that is key for active urease to deliver the nickel to the active center, and the binding between nickel and UreG played a critical role in transferring nickel from UreE to the urease (Yang et al. 2015; Yuen et al. 2017). As shown in Fig. 2A, both berberine chloride and epiberberine decreased the heat released when nickel was injected into the sample cell to combine with UreG, and larger heat changes were found in the present of epiberberine. In addition, berberine chloride slightly altered N value (nickel: UreG) and* K*_*D*_ value between nickel and UreG, while epiberberine decreased N value and increased *K*_*D*_ value significantly (*P* < 0.05) (Fig. 2B, C), indicating that binding affinity of nickel and UreG was weaker after adding the epiperberine, which was 10.8-fold weaker binding compared to that of berberine chloride.

### Alkaloids induced secondary structure changes of UreG

**Fig. 3:**
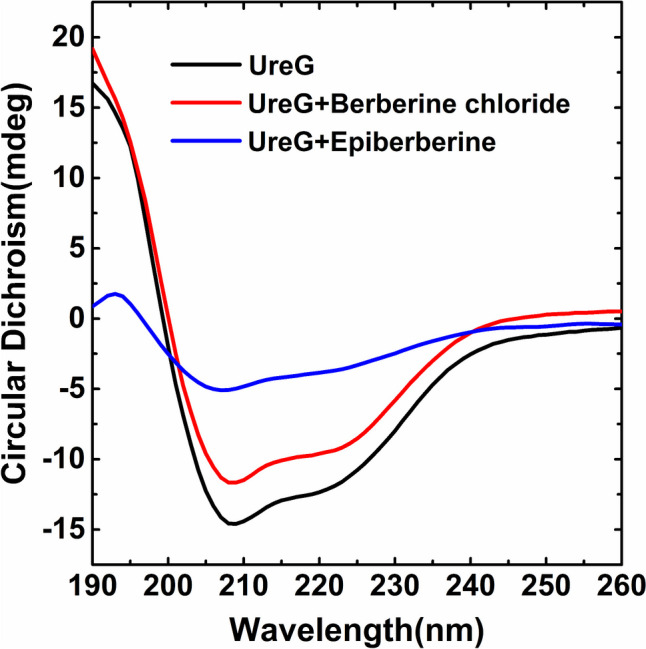
Effect of berberine chloride and epiberberine on second structure of UreG. Circular dichroism (CD) spectroscopy of UreG in the absence (black) or presence of berberine chloride (red) and epiberberine (blue)

We next investigated the effect of alkaloids on the secondary structure change of UreG, which was crucial for facilitating the transfer of nickel from UreGFH complex to the active site of urease (Nim et al. 2023). Figure 3 represented the CD spectra for UreG with different alkaloids, compared to the spectrum of UreG, that of adding alkaloids displayed a decrement in ellipticity (mdeg) at 208 and 222 nm, which was a signature of structure changes of α-helix. The overall spectral change of CD spectrum upon addition of epiberberine was greater than that of berberine chloride. The secondary structural content changes of UreG under different alkaloids are shown in Fig. S2, the reduced contents of α-helix and β-turn as well as increased contents of β-sheet and random coil were observed upon addition of alkaloids, in which berberine chloride caused slight changes, and epiberberine resulted in much larger changes in the proportions of α-helix and β-sheet, with ~ 56% decrease in the α-helix content and ~ 105% increasement in the β-sheet content. In summary, epiberberine induced larger variations in nickel-UreG binding and secondary structure of UreG compared to that of berberine chloride.

### Molecular docking of alkaloids towards UreG

**Fig. 4:**
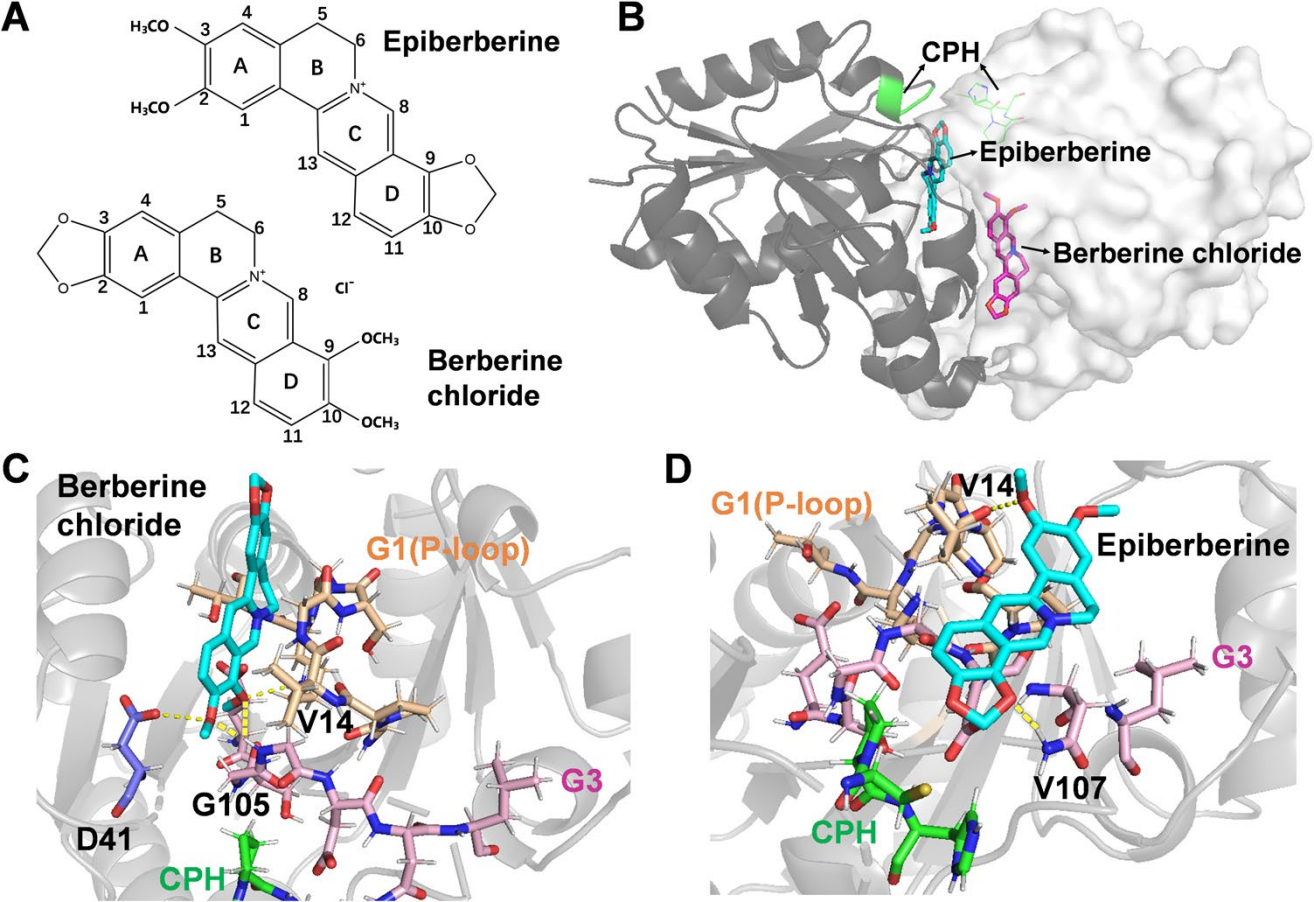
Binding mode of alkaloids with UreG. **A** The structures of berberine chloride and epiberberine. **B** Location diagram of berberine chloride and epiberberine in UreG dimer. **C** Interactions of berberine chloride with UreG. **D** Interactions of epiberberine with UreG. The structure of alkaloid is colored in cyan. Yellow dotted line represents the interaction between alkaloid and UreG. G1 (P-loop) motif, G3 motif, and CPH metal binding motif are colored orange, pink, and green, respectively

After confirming the overall effects of alkaloids on the binding between UreG and nickel as well as the secondary structure of UreG, we determined the molecular mechanism of UreG-alkaloid interactions using molecular docking. Epiberberine and berberine were two geometric isomers with the similar backbone structures, and the substituent groups were the same on different rings of A and D (Fig. 4A). The positions of berberine chloride and epiberberine in the UreG dimer are shown in Fig. 4B, and the UreG protomer on the right was used for receptor during molecular docking. Both berberine chloride and epiberberine were located at the dimer interface between two UreG protomers, while epiberberine was closer to the CPH nickel-binding motif, especially the D ring. As shown in Fig. 4C, D, the two alkaloids were located at the guanine nucleotide binding pocket, involving G1 motif (P-loop, orange color) and G3 motif (pink color). D ring of berberine chloride interacted with amino acid residues Asp41, Gly105, and Val14 and made a hydrogen bonding interaction with Gly105. Dimethylene group in the D ring of epiberberine was shown to form a hydrogen bond with Val107 residue in G3 motif, and methoxyl group in the A ring interacted with Val14 residue. In addition, we generated the binding affinities of UreG for berberine chloride and epiberberine with − 5.6 and − 5.7 kcal/mol, respectively. In general, the binding sites of alkaloids and UreG were located at the guanine nucleotide, which was key for the nickel insertion into active sites of urease (Fong et al. 2013). These induced changes by berberine chloride and epiberberine, including nickel-UreG binding and secondary structure of UreG, may be related to the binding residues of alkaloids and UreG.

### Effects of epiberberine on urease activity and the concentrations of NH_3_ and urea during ruminal microbial fermentation

**Fig. 5:**
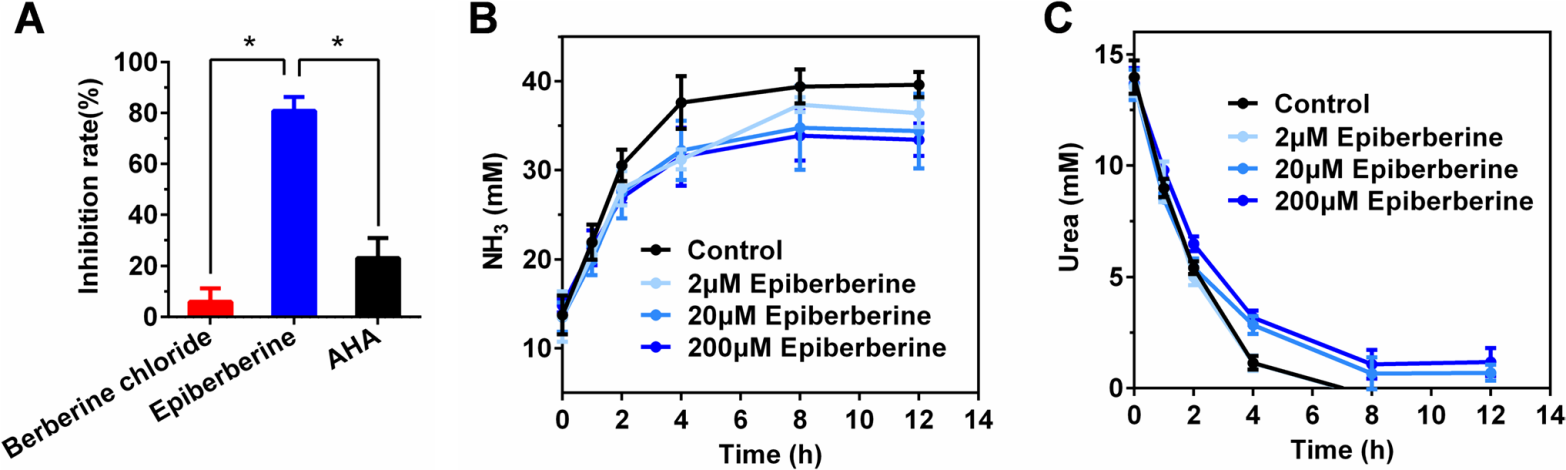
Effect of alkaloids on urease activity and ruminal microbial fermentation. **A** Inhibition rate of alkaloids and AHA on urease activity of ruminal microbial crude protein. Results are desired as means ± SD of triplicate tests. **B** Effect of epiberberine on the ammonia release during ruminal microbial fermentation. **C** Effect of epiberberine on the urea degradation during ruminal microbial fermentation. Each point represents means ± SD of triplicate tests

To get an insight of the possibility of berberine chloride and epiberberine for further developing as urease inhibitor, urease activities of the two alkaloids against ruminal microbial crude protein were measured, and the results are shown in Fig. 5A. Compared to positive control AHA, epiberberine significantly increased inhibition rate of urease activity against ruminal microbial crude protein (*P* < 0.05). Furthermore, the inhibitory potential of epiberberine was verified by ruminal microbial fermentation in vitro. The NH_3_ release and urea remaining through 12 h of incubation are shown in Fig. 5B, C. The NH_3_ concentration increased and urea content decreased along with the incubation time, and the concentration changes of NH_3_ and urea were more rapidly over the course of the first 4 h of incubation than that of 4 ~ 12 h of incubation. The rates of NH_3_ release and urea degradation tended to decrease with increasing dose of epiberberine. At 12 h of incubation, addition of 20 and 200 μM of epiberberine resulted in a decrease in NH_3_ concentration of 13.1% and 15.6%, respectively. Urea in control group and 2 μM epiberberine group was completely decomposed after 8 h of incubation. Taken together, epiberberine efficiently inhibited urease activity of ruminal microbial crude protein and slowed down the urea degradation in ruminal microbial fermentation in vitro, suggesting that epiberberine would be an effective inhibitor of rumen microbial urease.

## Discussion

In recent years, with the demand for natural foods rich in biological activities and the discovery of potential threat of antibiotics in the livestock industry, natural products from plants have attracted more and more attentions and interests. In view of the natural safety characteristics of *Rhizoma Coptidis* and effective effect in treating *Helicobacter pylori*-related gastrointestinal diseases, protoberberine alkaloids from *Rhizoma Coptidis* are considered as potential candidates of inhibitors against rumen microbial urease. In the study, epiberberine was identified as the most promising inhibitor of ruminal microbial urease by targeting UreG, and the effectiveness was further confirmed in the experiment of ruminal microbial fermentation.

The strategy of developing UreG as a target for design of urease inhibition was first proposed by Yang et al. (2018), and the main reason for targeting UreG was the key role of UreG in facilitating urease maturation, which mainly included the following three aspects. Firstly, delivering nickel to active center of apo-urease. Ni-UreE provided the nickel source for UreG by forming a complex UreE-UreG, and then Ni-UreG bound to UreFH to form a complex, delivering nickel to active center of apo-urease by the formation of a supercomplex apo-urease/UreFHG (Fong et al. 2013). Secondly, GTP hydrolysis. The final step of supercomplex releasing nickel into apo-urease was upon GTP hydrolysis by UreG. Lastly, GTP-dependent conformational changes. Nickel coordinated the residues of Cys66 and His68 in UreG. GTP hydrolysis induced conformational changes in UreG, disrupting the square-planar coordination by Cys66/His68 in the CPH motif and promoting the release of the nickel (Yang et al. 2015). In the study, considering the simplicity of GTPase activity detection, protoberberine alkaloids from *Rhizoma Coptidis* were preliminary selected by measuring the effect on UreG GTPase activity (the ability of UreG GTP hydrolysis) and then further identified by assessing several changes in the interaction of nickel towards UreG and secondary structure of UreG. There are many promising urease inhibitors, including hydroxamic acids, phosphoramidates, and urea derivatives. Up to now, AHA is only one commercially available inhibitor of urease approved by the Food and Drug Administration for the treatment of humans and animals (Kosikowska and Berlicki 2011), developing UreG as a new target would promote the development for the design of urease inhibitors.

*Rhizoma Coptidis* was commonly used in medicinal and food industries (Han et al. 2019), especially in traditional oriental medicine for the treatment of gastroenteritis; therefore, the main function compounds from *Rhizoma Coptidis*, including coptisine, berberine, palmatine, epiberberine, and jateorhizine, were regarded as promising inhibitor candidates of rumen microbial urease. In addition, salt selection is a wide technique used in the biological and pharmaceutical industries to enhance physicochemical properties of drug molecules, such as bioavailability, stability, and solubility (Dennany et al. 2015). It has been reported that half of all drug compounds are in medicinal therapy in the form of salt (Bharate 2021). In the study, protoberberine alkaloids, alkaloid hydrochlorides, and epiberberine from *Rhizoma Coptidis*, as well as AHA, were collected for preliminary selecting inhibitor by measuring their inhibition rates against GTPase activity. The results showed that alkaloid hydrochlorides had slightly higher inhibition rate than that of alkaloids themselves, and the inhibition rate of coptisine chloride was 2.5 times that of coptisine, indicating that alkaloid hydrochlorides will be a better choice for future applications. Notably, AHA, only one commercially available inhibitor of urease, had little effect on UreG GTPase activity, which indicated that AHA inhibited urease activity only by targeting its active center.

CPH metal binding motif from each protomer of UreG was juxtaposed to bind nickel, and epiberberine was closer to the CPH motif than berberine chloride. Epiberberine and berberine were two geometric isomers with the similar backbone structures, and the substituent groups were the same on different rings of A and D (Liu et al. 2020). For epiberberine, two methoxyl groups in the A ring were the polar systems of the alkaloid unit, and a dimethylene group in the D ring was the hydrophobic ring system. Jung et al. (2009) reported that the dimethylene group in the D ring of epiberberine contributed to inhibiting the activity of butyrylcholinesterase. In the study, the methoxyl group in berberine chloride and epiberberine interacted with amino acid residues Val14; the dimethylene group in berberine chloride did not bind with any of the residues in UreG, while that of in epiberberine contact Val107 residue in G3 motif. The better inhibitory potential of epiberberine may be related to the presence of the dimethylene group in the D ring.

We examined the effects of berberine chloride, epiberberine, and AHA on urease activity against ruminal microbial crude protein and found that the inhibitory rate of epiberberine was significantly higher than that of berberine chloride and AHA (*P* < 0.05). Yang et al. (2018) showed that bismuth significantly inhibited urease activity in live bacterial cells and had little inhibitory effect on extracted *Helicobacter pylori* urease, indicating that bismuth inhibited activity of *H. pylori* urease only by disrupting urease maturation in bacteria, rather than by combining with the active sites of urease. Li et al. (2016) found berberine failed to show any inhibitory activity against urease in vitro; our study showed that berberine chloride inhibited UreG GTPase activity and urease activity of ruminal microbial crude protein. The inhibition mechanism of berberine may be resembled similar to that of bismuth, which only perturbed the nickel deliver by UreG. In the study, AHA had little inhibitory effect on UreG GTPase activity, and the inhibition rate against ruminal microbial crude protein reached 40%, suggesting that the active sites of urease were possibly responsible for the inhibition of AHA. Epiberberine has been revealed to inhibit urease activity of *Helicobacter pylori* by binding to the sulfhydryl group at the active center of urease. Combined with the results of this study, the inhibition mechanism of epiberberine may related to both active centers of urease and urease maturation, which may be the reason why the inhibitory rate of epiberberine against ruminal microbial crude protein was significantly higher than that of both berberine chloride and AHA.

The safety of berberine, palmatine, coptisine, and epiberberine was evaluated by Yi et al. (2013). The cell assay found that the cytotoxicity of epiberberine was the lowest with the highest IC_50_ value of 120.6 mg/mL. In the acute toxicity experiment, the median lethal dose of palmatine and epiberberine was 1533.7 and 1360.0 mg/kg, respectively, which were larger than the other two alkaloids. The results of the sub-chronic toxicity research found that there was no abnormality in all clinical signs after the oral administration of the 4 alkaloids. In addition, the oral bioavailability of epiberberine in rats was 14.5%, which was much higher than that of berberine (< 1%) (Chen et al. 2017). Notably, epiberberine was not detected in rat samples, including urine, feces, and bile, after oral administration of *Rhizoma Coptidis* extract (6.7% epiberberine in the extract) (Feng et al. 2020). These results indicated that developing epiberberine as a safe urease inhibitor would be superior to that of any other alkaloids from *Rhizoma Coptidis*. Furthermore, epiberberine inhibited NH_3_ release and urea degradation during ruminal microbial fermentation, confirming the effectiveness of epiberberine in inhibiting activity of rumen microbial urease.

## Supplementary Information

Below is the link to the electronic supplementary material.

Supplementary file1 (PDF 194 KB)

## Data Availability

The datasets generated during and/or analyzed during the current study are available from the corresponding author on reasonable request.
