# Supplementary material for: Epiberberine: a potential rumen microbial urease inhibitor to reduce ammonia release screened by targeting UreG
Source: Appl Microbiol Biotechnol. 2024 Apr 8;108:289. doi: 10.1007/s00253-024-13131-4 (PMC11001712; doi:10.1007/s00253-024-13131-4)
Supplement: Supplementary file 1 [file 253_2024_13131_MOESM1_ESM.pdf]

Applied Microbiology and Biotechnology

Epiberberine: a potential rumen microbial urease inhibitor to reduce ammonia release screened by targeting UreG

Xiaoyin Zhang · Zhanbo Xiong · Yue He · Nan Zheng · Shengguo Zhao* · Jiaqi Wang*

State Key Laboratory of Animal Nutrition and Feeding, Institute of Animal Sciences, Chinese Academy of Agricultural Sciences, Beijing 100193, China

*** Correspondence:** Shengguo Zhao, zhaoshengguo1984@163.com; Jiaqi Wang, jiaqiwang@vip.163.com

Supplemental materials

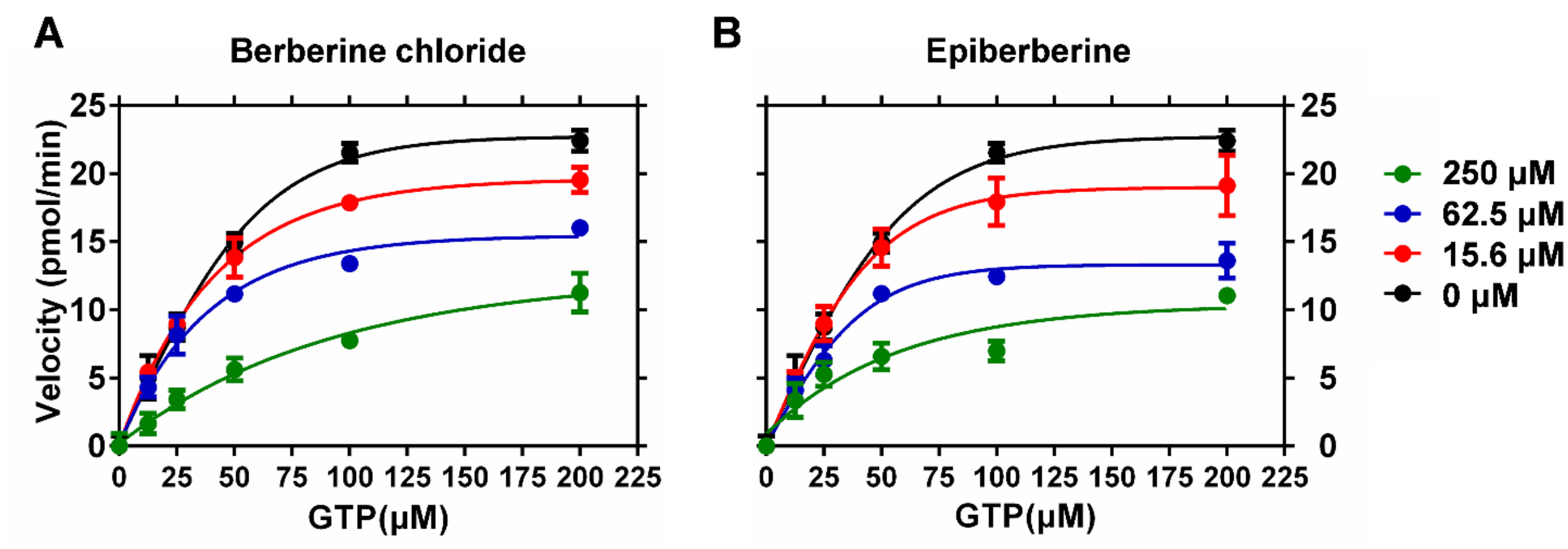

Fig. S1 Kinetic study of UreG inhibition by berberine chloride (A) and epiberberine (B). The various concentrations of GTP substrate are 0, 12.5, 25, 50, 100, and 200 μM, and that of berberine chloride and epiberberine are 0, 15.6, 62.5, and 250 μM. Each point represents means ± SD of triplicate tests.

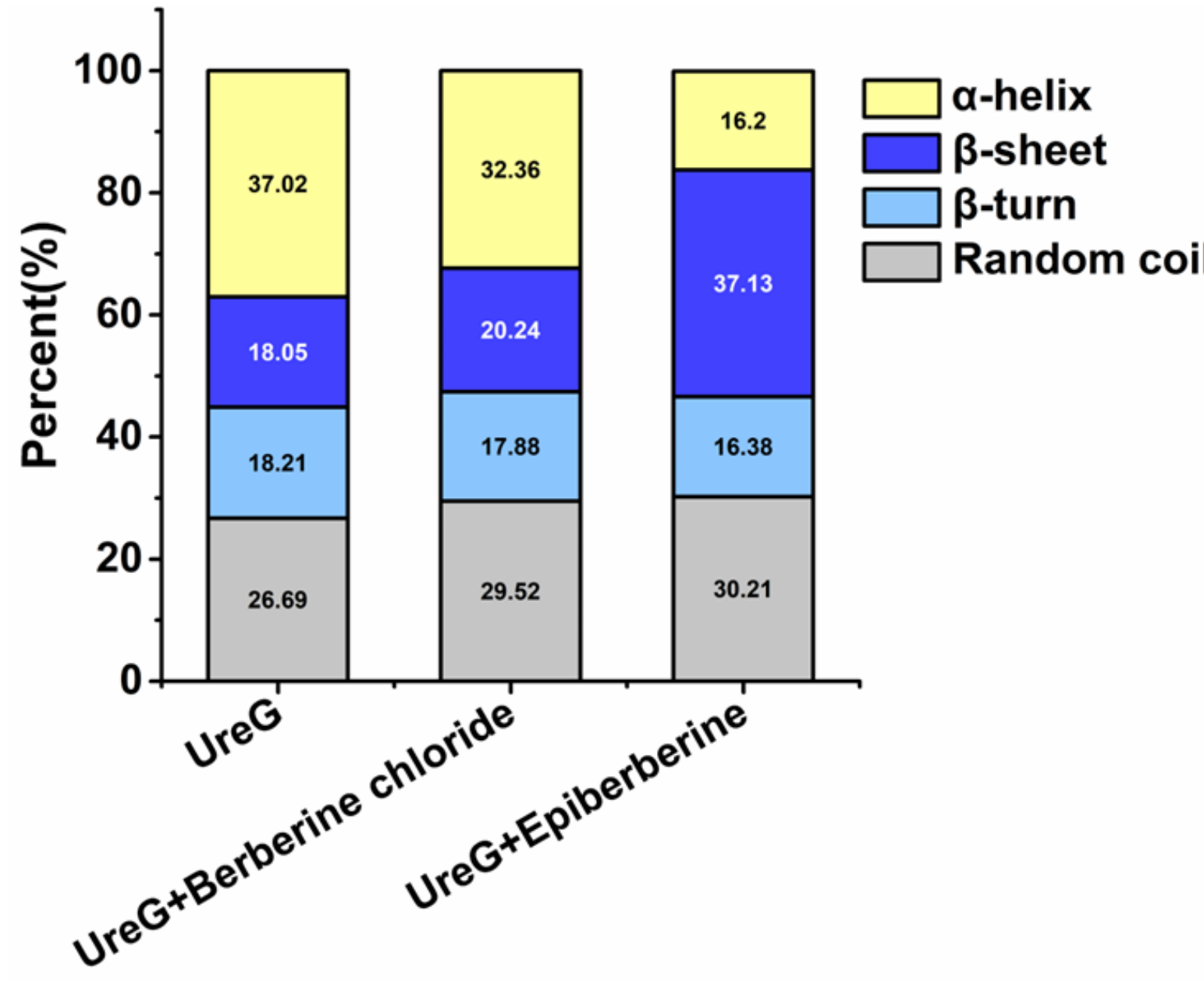

Fig. S2 Effect of berberine chloride and epiberberine on the proportions of second structure of UreG.
